# Diagnostic and prognostic value of antibodies against chimeric fibrin/filaggrin citrullinated synthetic peptides in rheumatoid arthritis

**DOI:** 10.1186/ar2802

**Published:** 2009-09-02

**Authors:** Raimon Sanmartí, Eduard Graell, Maria L Perez, Guadalupe Ercilla, Odette Viñas, Jose A Gómez-Puerta, Jordi Gratacós, Alejandro Balsa, Maria J Gómara, Marta Larrosa, Juan D Cañete, Isabel Haro

**Affiliations:** 1 Rheumatology Service, Hospital Clínic of Barcelona, IDIBAPS, Villarroel 170, 08036 Barcelona, Spain; 2 Rheumatology Unit, Hospital Universitari Parc Taulí of Sabadell, Parc Taulí s/n 08208 Sabadell, Barcelona, Spain; 3 Unit of Synthesis and Biomedical Applications of Peptides IQAC-CSIC, Jordi Girona 18-26, 08034, Barcelona, Spain; 4 Immunology Service, Hospital Clínic of Barcelona, Villarroel 170, 08036 Barcelona, Spain; 5 Rheumatology Service, Hospital Universitario La Paz, Paseo de la Castellana 261, 28046 Madrid, Spain

## Abstract

**Introduction:**

Evidence suggests that citrullinated fibrin(ogen) may be a potential *in vivo *target of anticitrullinated protein/peptide antibodies (ACPA) in rheumatoid arthritis (RA). We compared the diagnostic yield of three enzyme-linked immunosorbent assay (ELISA) tests by using chimeric fibrin/filaggrin citrullinated synthetic peptides (CFFCP1, CFFCP2, CFFCP3) with a commercial CCP2-based test in RA and analyzed their prognostic values in early RA.

**Methods:**

Samples from 307 blood donors and patients with RA (322), psoriatic arthritis (133), systemic lupus erythematosus (119), and hepatitis C infection (84) were assayed by using CFFCP- and CCP2-based tests. Autoantibodies also were analyzed at baseline and during a 2-year follow-up in 98 early RA patients to determine their prognostic value.

**Results:**

With cutoffs giving 98% specificity for RA versus blood donors, the sensitivity was 72.1% for CFFCP1, 78.0% for CFFCP2, 71.4% for CFFCP3, and 73.9% for CCP2, with positive predictive values greater than 97% in all cases. CFFCP sensitivity in RA increased to 80.4% without losing specificity when positivity was considered as any positive anti-CFFCP status. Specificity of the three CFFCP tests versus other rheumatic populations was high (> 90%) and similar to those for the CCP2. In early RA, CFFCP1 best identified patients with a poor radiographic outcome. Radiographic progression was faster in the small subgroup of CCP2-negative and CFFCP1-positive patients than in those negative for both autoantibodies. CFFCP antibodies decreased after 1 year, but without any correlation with changes in disease activity.

**Conclusions:**

CFFCP-based assays are highly sensitive and specific for RA. Early RA patients with anti-CFFCP1 antibodies, including CCP2-negative patients, show greater radiographic progression.

## Introduction

Anti-citrullinated protein/peptide antibodies (ACPAs) are considered the most specific serologic markers of rheumatoid arthritis (RA) [1]. Although the sensitivity is similar to that of rheumatoid factor (RF)--the only antibody included in the American College of Rheumatology (ACR) classification criteria--ACPAs have a higher specificity [2]. ACPAs recognize proteins or peptides with arginine residues converted to citrulline by a posttranslational modification and have diagnostic and prognostic significance [3]. Recent studies have demonstrated that ACPAs are the strongest serum marker associated with future RA progression in patients with recent-onset arthritis [4,5] and radiographic progression in ACPA-positive patients with early RA is greater than that in ACPA-negative patients [6-8]. ACPAs also have been linked to certain genetic and epidemiologic characteristics such as the HLADRB04 genotype [9] and smoking [10].

ACPAs can be detected by using enzyme-linked immunosorbent assays (ELISAs) with different citrullinated protein or peptide substrates. The most widely used in clinical practice is the cyclic citrullinated peptide 2 assay (CCP2) [1-3]. The cyclic peptide(s) in the CCP2 have no homology with known proteins [1] and improved the sensitivity of the first test to become available based on a synthetic citrullinated peptide derived from filaggrin (CCP1) [11]. More recently, other ELISA tests using citrullinated mutated vimentin [12], citrullinated human fibrinogen [13], or third-generation citrullinated peptides (CCP3) [14] with useful diagnostic and prognostic properties have been developed. Although most ACPA-based tests seem to have similar diagnostic yields, discrepancies may arise, probably because of the different antigen sources used [15].

Although original assays used citrullinated peptides derived from human filaggrin, this epithelial protein is not expressed in synovial tissues and so is probably not the *in vivo *target of these autoantibodies. Several proteins present in inflamed rheumatoid synovium, such as type I collagen, vimentin, α-enolase, and fibrin(ogen), can be citrullinated [16]. Citrullinated fibrin has been identified as a potential synovial target for ACPA [17], and two citrullinated fibrin-derived peptides in the α and β chains of human fibrin have been found to be the main antigen substrates for these antibodies [18]. We previously demonstrated the special ability and specificity of a citrullinated peptide sequence of α-fibrin ([Cit^621,627,630^]α-fibrin(617-631)) to recognize autoantibodies in RA sera [19]. Three cyclic citrullinated peptides derived from these regions of α-fibrin were found to recognize serum autoantibodies in RA in a subsequent study [20]. Further interesting results were obtained with a chimeric fibrin/filaggrin citrullinated peptide (CFFCP1) containing an α-fibrin peptide and the cyclic filaggrin peptide, cfc-1cyc, which forms the basis of the commercial CCP1 test. The noncommercial ELISA test using this chimeric citrullinated synthetic peptide was more sensitive than the commercial one using CCP1 (82% vs. 65.8%) in an RA population and reacted with some CCP2-negative sera [20].

The aim of the present study was to evaluate the diagnostic yield of three ELISA tests based on this CFFCP1 peptide and two new synthetic chimeric fibrin/filaggrin peptides (CFFCP2, CFFCP3) and to compare their sensitivity and specificity in RA and other disease groups with the commercial CCP2 test. The prognostic value of these chimeric ACPAs also was studied in a cohort of patients with early RA.

## Materials and methods

### Diagnostic yield

#### Patients

The study included five different populations. The adult RA population comprised 322 patients, all of whom fulfilled the 1987 ACR classification criteria. Of these, 70.4% were positive for RF, and 134 had early RA (< 2 years of disease duration). Seventy-eight patients were receiving biologic therapy (tumor necrosis factor (TNF)-α antagonist) at the time of serologic determination. Four different control groups were included: 307 healthy blood donors, 119 patients with systemic lupus erythematosus (SLE) according to ACR criteria, 133 patients with psoriatic arthritis (PsA) fulfilling the Wright and Moll criteria, and 84 patients with chronic hepatitis C virus (HCV) infection (confirmed by nucleic acid testing and antibody test results).

### Prognostic value

#### Patients

All 134 patients with early RA from the study of diagnostic value were included in a prospective study of prognostic factors in early RA. Two years of follow-up data were available for 98 of 134 patients. Results for radiographic and clinical evolution in this early RA population have been published elsewhere [21,22]. All were outpatients attending the rheumatology departments of the Hospital Clínic of Barcelona or the Hospital Parc Taulí of Sabadell, Spain.

#### Study design and follow-up

All patients were treated according to a therapeutic protocol by using a step-up approach with early introduction of DMARDs together with very low doses of glucocorticoids (methylprednisolone, 4 mg/day) [21,22]. After the first year of therapy, patients were treated according to the criteria of the attending physician, but with an aggressive approach; other DMARDs were added when the response to previous DMARDs was poor. Biologic therapy was started in a few patients with a poor response to DMARD therapy. Demographic characteristics and disease duration were recorded at study entry. At baseline and at 6, 12, 18, and 24 months, disease activity was measured by using the index of disease activity score in 28 joints (DAS28) [23], and disability, by using the modified Health Assessment Questionnaire (mHAQ) [24]. Serum rheumatoid factor (RF) was measured with nephelometry (BNII, Siemens; normal values, < 25 UI/ml). Radiographs of hands and feet were taken at 0, 12, and 24 months. The modified Larsen method [25] was used to evaluate radiographic damage, as previously described [21]. Each participant signed a written informed consent. Both studies were approved by the Ethics Committee of the Hospital Clinic of Barcelona.

#### Chimeric fibrin/filaggrin synthetic peptides

The following chimeric α-fibrin-filaggrin citrullinated peptides were synthesized:

CFFCP1: [Cit^630^]αfibrin(617-631)-*S*^306^, *S*^319^cyclo [Cys^306,319^, Cit^312^]filaggrin(304-324)

CFFCP2: [Cit^627,630^]αfibrin(617-631)-*S*^306^, *S*^319^cyclo [Cys^306,319^, Cit^312^]filaggrin(304-324)

CFFCP3: [Cit^621,630^]αfibrin(617-631)-*S*^306^, *S*^319^cyclo [Cys^306,319^, Cit^312^]filaggrin(304-324)

The chimeric linear sequences were synthesized in the solid phase with subsequent cyclization in solution by forming a disulfide bridge, as previously described for CFFCP1 [20].

Crude peptides were purified with preparative high-performance liquid chromatography (HPLC). The identity of the purified peptides was confirmed with electrospray mass spectrometry, and their purity was determined with analytic HPLC. The overall yield from peptide resins was 30% to 45%.

#### ELISA

Peptide sequences were coupled covalently to ELISA microtiter plates (Costar Corp., Cambridge, MA) as described previously [20]. All sera were tested in duplicate. Control sera were also included to monitor inter- and intraassay variations. Sera also were analyzed by using second-generation CCP2-based ELISA (Immunoscan, Eurodiagnostica, distributed by Diasorin Madrid, Spain). For patients included in the prognostic analysis, serum samples were obtained at baseline and after 1 and 2 years.

### Statistical methods

Cutoff values for chimeric and commercial ELISA tests were selected with ROC analysis to give a specificity of 98% for RA versus healthy blood donors. The association between categorized anti-citrullinated antibodies and other qualitative variables was tested by using the χ^2 ^test and Fisher's Exact test, as applicable. Sensitivity, specificity, and positive and negative predictive values (PPVs and NPVs) of the ELISA tests were calculated. To analyze anti-CFFCP and anti-CCP2 status with relevant quantitative variables, the *t *test and analysis of variance were used unless conditions for such tests were not met, in which case the Mann-Whitney *U *and Kruskal-Wallis tests were used. Changes from baseline of the different autoantibodies titers after 1 and 2 years of follow-up were analyzed by using the *t *test for paired samples. The correlation between different citrullinated peptide antibodies and the association between antibody values and quantitative clinical variables were tested with Pearson's correlation or Spearman's rank correlation, as applicable. Comparison and analysis of ROC curves were performed with MedCalc v7.6 software. Statistical analyses were performed by using SPSS 16.0. All *P *values were two sided, and *P *< 0.05 was considered statistically significant.

## Results

### Diagnostic value of anti-CFFCP antibodies

For the different ELISA tests investigated in this study, the area under the ROC curves (RA vs. healthy blood donors) was not significantly different: CFFCP1, 0.926 (95% CI, 0.905-0.947); CFFCP2, 0.953 (95% CI, 0.938-0.969); CFFCP3, 0.925 (95% CI, 0.903-0.947), and CCP2, 0.940 (95% CI, 0.921-0.959). Cutoff values for each ELISA test calculated according this ROC curve analysis were CFFCP1, 0.241 optic density units (ODUs); CFFCP2, 0.229 ODUs; CFFCP3, 0.280 ODUs; and CCP2, 30 IU/L. For cutoff values giving a specificity of 98% with respect to blood donors, the sensitivity of the noncommercial ELISAs was 72.1% for CFFCP1, 78% for CFFCP2, and 71.4% for CFFCP3, with the PPV greater than 97% in all three tests and the NPV between 76.7% and 80.9%. Similar results were obtained by using the commercial CCP2 test (sensitivity, 73.9%; PPV, 97.6%; and NPV, 78.2%). The sensitivity of the three CFFCP tests was significantly lower in RF-negative RA compared with RF-positive RA (Table 1). As expected, serum titers of all citrullinated peptide antibodies were higher in RF-positive RA compared with RF-negative RA. However, patients with citrullinated peptide antibody titers above cutoff showed no differences in mean serum levels between RF^+ ^and RF^- ^RA patients.

**Table 1:** Sensitivity of commercial CCP2 test and noncommercial CFFCP enzyme-linked immunosorbent assays in different rheumatoid arthritis populations and control groups

Population	*n*	CFFCP1	CFFCP2	CFFCP3	CCP2
Rheumatoid arthritis	322	232 (72%)	251 (78%)	230 (71.4%)	238 (73.9%)
RF^+^	226	188 (83.2%)	199 (88.1%)	192 (85%)	199 (88.1%)
RF^-a^	95	43 (45.3%)	52 (54.7%)	38 (40%)	39 (41.1%)
Early	134	102 (76.1%)	104 (77.6%)	99 (73.9%)	99 (73.9%)
Anti-TNF-α treatment	78	56 (71.8%)	62 (79.5%)	61 (78.2%)	62 (79.5%)
Healthy blood donors	307	6 (2%)	6 (2%)	6 (2%)	6 (2%)
Systemic lupus erythematosus	119	11 (9.2%)	11 (9.2%)	9 (7.6%)	9 (7.6%)
Psoriatic arthritis	133	6 (4.5%)	13 (9.8%)	2 (1.5%)	4 (3%)
Hepatitis C virus infection	84	12 (14.3%)	2 (2.4%)	2 (2.4%)	1 (1.2%)

^a^*P *< 0.0001 for all autoantibodies in comparison with RF+ rheumatoid arthritis. In one RA patient, RF status was not available. RF = rheumatoid factor; TNF = tumor necrosis factor.

**Figure 1:**
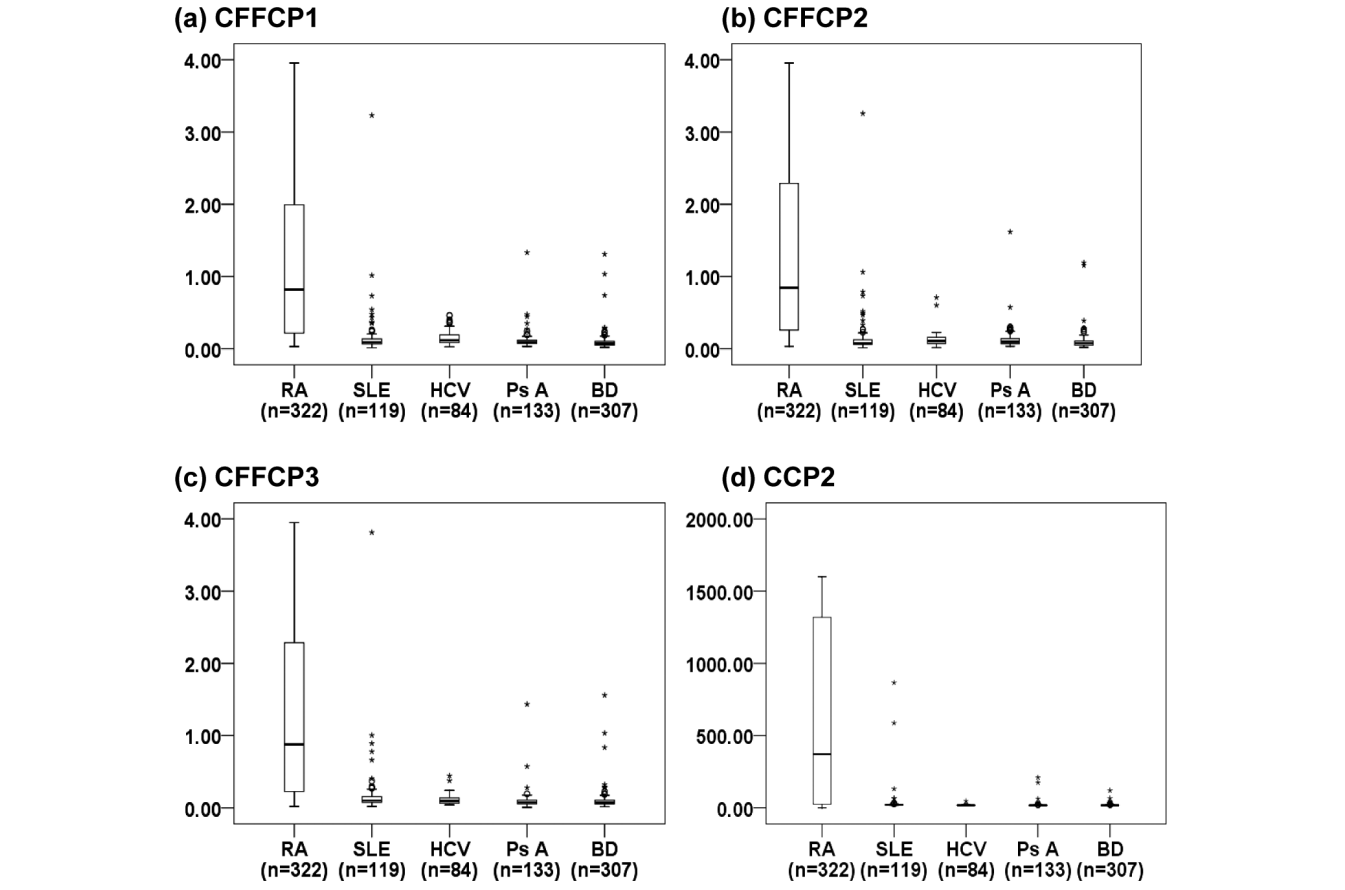
Titers of autoantibodies against the chimeric citrullinated fibrin/filaggrin peptides anti-CFFCP1, anti-CFFCP2, anti-CFFCP3, and against cyclic citrullinated peptide CCP2 in RA and control populations. Boxes represent interquartile range and median. Whiskers represent values outside the interquartile range. Single points represent outliers and extreme values. RA = rheumatoid arthritis; SLE = systemic lupus erythematosus; HCV = hepatitis C virus infection; PsA = psoriatic arthritis; BD = blood donor.

No significant differences in sensitivity were found between the different assays according to the RA population studied (Table 1). In the control populations, positive results were observed in only a small proportion of patients in each type of ELISA test (Figure 1), and these patients had low levels (Table 2). In the SLE group, positive results were observed in 7.6% to 9.2% of sera in the CFFCP tests and commercial CCP2 tests. Most patients with SLE and positive ELISA tests had antibody titers just above normal. In the PsA and HCV groups, fewer than 4.5% of patients had positive anti-CFFCP or anti-CCP2 status, except for anti-CFFCP2 status in PsA (9.8%) and anti-CFFCP1 status in HCV (14.3%).

**Table 2:** Serum levels of anti-CFFCP and anti-CCP2 antibodies in the sera of RA and non-RA populations with values above the cut-off point

		CFFCP1		CFFCP2		CFFCP3		CCP2	
					
	*n*	No. positive	Mean (Std)	No. positive	Mean (Std)	No. positive	Mean (Std)	No. positive	Mean (Std)
RA	322	232	1.58 (1)	251	1.61 (1.08)	230	1.70 (1.04)	238	842 (605)
SLE	119	11	0.73 (0.86)	11	0.78 (0.86)	9	0.95 (1.11)	9	205 (305)
HCV	84	12	0.34 (0.08)	2	0.66 (0.08)	2	0.41 (0.05)	1	46
PsA	133	6	0.52 (0.41)	13	0.39 (0.38)	2	1 (0.61)	4	120 (84)
BD	307	6	0.66 (0.45)	6	0.59 (0.45)	6	0.72 (0.52)	6	67 (37)

No = total number of patients; No. positive = number of positive patients; Std = standard deviation. Statistical differences were observed between RA and non-RA serum levels (*P *< 0.001).

A very good correlation between the three types of CFFCP test was observed in RA (*R *values between 0.96 and 0.98; *P *< 0.0001), and a good correlation also was observed between CFFCP tests and the CCP2 test (*R *values between 0.87 and 0.89; *P *< 0.0001), although discrepancies were observed in some RA sera. Positive results with CFFCP1, CFFCP2, and CFFCP3 were observed in 14.3%, 27.4%, and 9.5% of sera from anti-CCP2-negative patients, respectively, and anti-CCP2-positive status was observed in 20%, 28.9%, and 13.9% of patients negative for anti-CFFCP1, CFFCP2, and CFFCP3, respectively. In 1.9% of sera, anti-CCP2 status was positive, although anti-CFFCP status was negative. In addition, 1.9% of patients positive in the three CFFCP tests were negative with CCP2 ELISA. Discrepant cases tended to be associated with near-normal serum titers. When positivity was considered as one or more positive anti-CFFCP status, sensitivity increased to 80.4% whereas specificity remained high (97.7%).

### Prognostic value of anti-CFFCP antibodies

#### Demographic and clinical characteristics and disease course in the early RA population

At study entry, the mean age of the 98 patients (81.6% women) with early RA was 54.6 ± 14.8 years, and the disease duration was 9.1 ± 5.9 months. Rheumatoid factor was positive in 72.4% of patients. The mHAQ at baseline was 1 ± 0.5. DAS28 decreased significantly from a mean of 5.72 ± 0.9 at baseline to 3.72 ± 1.23 and 3.45 ± 1.23 at 1 and 2 years of follow-up, respectively. Remission (DAS28 < 2.6) was achieved by 23.7% and 30.9% of patients at month 12 and 24, respectively. The mean Larsen score progressed from 1.3 ± 2.7 at study entry to 6.0 ± 9.4 at the end of follow-up.

#### Anti-CFFCP antibodies in early RA

Serum levels of the three anti-CFFCP antibodies and anti-CCP2 antibodies were obtained at baseline in all 98 patients. Positive anti-CFFCP1, anti-CFFCP2, and anti-CFFCP3 status was reported in 70%, 73%, and 76% of patients, respectively, and anti-CCP2 status, in 73%.

Serial serum measurements of the different autoantibodies were made in the majority of patients at baseline and during the follow-up. In 56 patients, all four tests were available at baseline and at 1 and 2 years of follow-up. CFFCP status remained stable in most patients during the follow-up, but some patients became positive (2.3% to 12.8%) and even more became negative (12.2% to 21.7%). Negative conversion occurred mainly in patients with serum levels just above the normal range for both the CCFCP test and the commercial CCP2 test. Mean serum levels of the different anti-CFFCP and anti-CCP2 antibodies tended to decrease during the follow-up, although the difference was statistically significant only for anti-CFFCP2 and anti-CCP2 status at 1 year (data not shown).

#### Anti-CFFCP antibodies and disease activity

**Figure 2:**
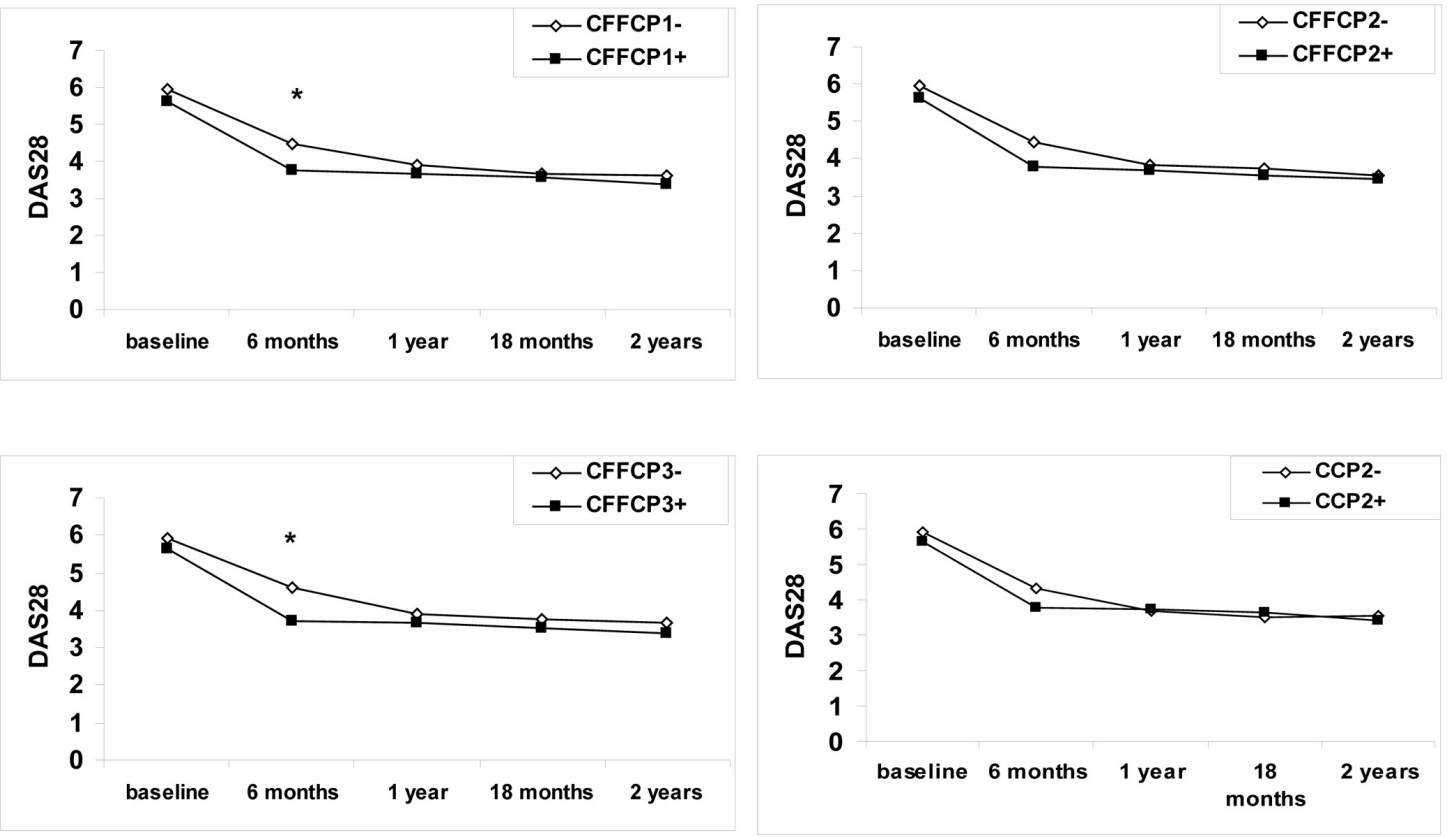
Comparative changes in the disease-activity score DAS28 during the 2 years of follow-up in early RA patients who are positive and negative for anti-CFFCP and anti-CCP2 at baseline. Anti-CFFCP1 (n = 101), anti-CFFCP2 (n = 98), anti-CFFCP3 (n = 98), and anti-CCP2 (n = 109). Mean values are presented. *P < 0.05.

At baseline, disease activity measured by the DAS28 score was similar in patients with and without anti-CFFCP1, anti-CFFCP2, and anti-CFFCP3 antibodies and those with and without anti-CCP2 antibodies. The DAS28 score decreased significantly in positive and negative patients, but differences were not significant at 1 and 2 years (Figure 2). No correlation between the variations in anti-CFFCP antibody titers and different measures of disease activity at 1 and 2 years was found, except in the case of anti-CFFCP3 titer and the swollen-joint count at 2 years (*P *= 0.04). The change in DAS28 at 2 years (*P *= 0.04) and change in the swollen-joint count at 1 and 2 years (*P *= 0.03 and *P *= 0.001) correlated with variations of the CCP2 test at 2 years, but the correlations disappeared after Bonferroni correction.

#### Anti-CFFCP antibodies and radiographic damage

**Figure 3:**
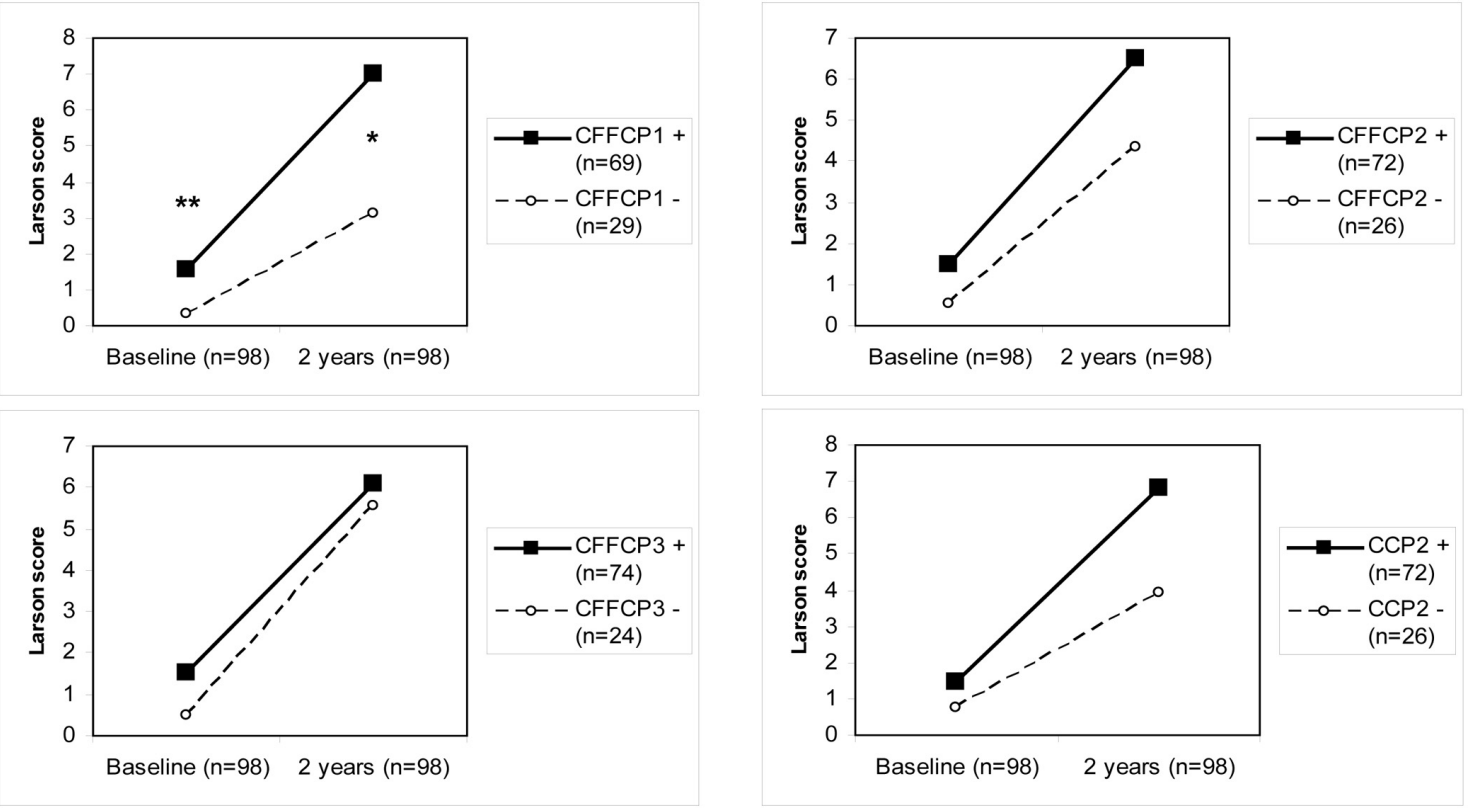
Radiographic progression (Larsen scores) and anti-CFFCP and anti-CCP2 status in patients with early RA after 2 years of follow-up. *P < 0.01. **P < 0.05.

Baseline radiographic damage measured by the Larsen score was significantly higher in patients with CFFCP1 antibodies than in those without. Higher scores were also observed in patients with positive anti-CFFCP2, anti-CFFCP3, and anti-CCP2 status than those with negative status, but these differences were not statistically significant. After 24 months, significantly higher Larsen scores were observed only for positive anti-CFFCP1 status versus negative status, although a clear nonsignificant trend was observed also for positive anti-CCP2 status (Figure 3). Mean Larsen scores at baseline and at 2 years were higher in the small group of patients negative for anti-CCP2 antibodies and positive for anti-CFFCP1 antibodies (n = 8; mean ± SD, 1.6 ± 2.2 and 6.4 ± 10.9, respectively) than were those observed in patients negative for both autoantibodies (n = 22; mean ± SD, 0.4 ± 1.2 and 3.0 ± 6.0, respectively) and similar to those observed in patients positive for both autoantibodies (n = 65; mean ± SD, 1.6 ± 3.1 and 7.0 ± 10.9, respectively).

## Discussion

This study analyzed the diagnostic and prognostic properties of three new ELISA tests for RA to detect ACPA. These noncommercial assays, which use synthetic chimeric cyclic citrullinated peptides from filaggrin and the α chain of fibrin as antigenic substrates, showed a high sensitivity and specificity for RA in comparison with healthy controls and those with other chronic diseases. Furthermore, in a group of early RA patients, these antibodies were associated with poor radiographic outcome.

Synovitis in RA is characterized by excessive generation and breakdown of fibrinogen [26]. Citrullinated fibrinogen may participate in the inflammatory process in RA, affecting the balance between coagulation and fibrinolysis [27] and acting as an important autoantigen [28]. Recent data on the arthritogenic properties of citrullinated fibrinogen in a mouse model of arthritis [29] and the presence of immune complexes containing citrullinated fibrinogen in the sera and synovium of RA point to a direct role in the pathogenesis of rheumatoid synovitis [30]. An *in vitro *human model demonstrated the inflammatory potential of ACPA-containing immune complexes of citrullinated fibrin, inducing TNF-α secretion by macrophages [31]. Together, these findings reinforce the role of citrullinated fibrinogen in RA pathogenesis and suggest that this protein could be an important *in vivo *target of the ACPAs present in RA sera and therefore an optimal antigenic substrate for the determination of ACPAs.

We analyzed the sensitivity and specificity of CFFCP1 in a large group of RA patients and other well-defined control groups. We also included two new synthetic chimeric peptides also derived from filaggrin and the α chain of fibrin (CFFCP2 and CFFCP3). The ELISA assays for chimeric citrullinated peptides showed a high sensitivity (71.4% to 78.0%) for RA diagnosis, similar to that seen for the CCP2-based test (73.9%), by using cutoff values yielding 98% specificity with respect to healthy blood donors. The sensitivity of CFFCP is similar in patients with early RA and in those with established disease, and lower in RF-negative patients, as previously reported [32].

As expected, the specificity of anti-CFFCP antibodies was not as high when compared with other chronic diseases, especially SLE, in which up to 9.2% of sera yielded positive results, as observed in our population and in other studies [33,34]. However, antibody levels were low in most SLE patients with positive results compared with RA patients. The different anti-CFFCP antibodies were also detected in a small number of patients with PsA or HCV infection.

As previously described by our group [19,20], not all citrullinated fibrin-derived peptides are equally immunoreactive with ACPA, reflecting the importance of the aminoacyl environment of citrulline residues and the correct balance of Arg/Cit residues within the synthetic molecule. This probably influences the adoption of a preferred conformation with enhanced capacity for binding to autoantibodies present in RA patients. Of all the α-fibrin-related peptides we analyzed, those spanning the region from 617 to 631, with a citrullyl residue at the 630 position, were clearly the most reactive [20]. Thus, in agreement with the results reported by Sebagg *et al*. [18], the Cit^630^-bearing peptides from α-fibrin could represent a good molecular mimic of the ACPA epitopes targeted *in vivo*. Particularly good results were obtained by combining two peptide units of different citrullinated proteins. In particular, CFFCP1, CFFCP2, and CFFCP3, the three 15-mer Cit^630^-bearing peptides from α-fibrin covalently coupled to the cyclic filaggrin peptide, which constitutes the CCP1 test [35], have been shown to be the most reactive ACPA epitopes [20].

The CCP2-based test was very specific and highly sensitive for RA, with a higher sensitivity than that of CCP1 [36]. Our results showed a similar sensitivity, specificity, and predictive values for the diagnosis of RA compared with the commercial CCP2 test. A similar diagnostic yield was described for CCP2 and anti-citrullinated fibrinogen in a study with an ELISA test containing human fibrinogen as the antigenic epitope [37]. A very strong correlation was found between the three anti-CFFCP antibodies and between anti-CFFCPs and anti-CCP2 antibodies, although a significant proportion of sera showed discrepancies, mainly in patients with antibodies near the cut-off values. Our studies also found discordant results when different commercial ELISA tests were compared, with the main explanation being the different types of antigenic peptide/proteins used [15,38]. The sensitivity of the presence of any of the three anti-CFFCP antibodies increased to 80.5%, 6.6% higher than the commercial CCP2-based test, with no loss of specificity.

Studies have shown that ACPAs are associated with a poor disease outcome. Most of these studies used the commercial CCP1- or CCP2-based tests [6-8], but some also used antibodies against citrullinated human fibrin(ogen) [13,39], and mutated vimentin [12,40] has been associated with radiographic damage. In our cohort with early RA, modified Larsen scores at baseline and 2 years after starting DMARD therapy were higher in patients with positive baseline values of anti-CFFCPs, with significant differences at both time points for anti-CFFCP1 antibodies. A clear but nonsignificant trend also was observed for anti-CFFCP2, anti-CFFCP3, and anti-CCP2 status. Patients with positive anti-CFFCP and negative anti-CCP2 status had a radiographic progression similar to that observed in patients positive for both autoantibodies, indicating that these autoantibodies may be a markers of poor radiographic outcome in a subgroup of patients who are anti-CCP2 negative; however, the subgroups were too small to draw definitive conclusions.

Despite radiographic progression, patients with and without baseline anti-CFFCP antibodies and CCP2 followed a similar clinical course, according to DAS28. Short follow-up could explain why anti-CFFCP status at baseline does not correlate with disease activity, because some studies found a more-severe disease course in patients with ACPA, primarily after 2 years of follow-up [41,42].

As observed with anti-CCP2 antibodies [43] and antibodies against mutated vimentin in early RA [40], reduced anti-CFFCP antibody titers were observed in our cohort during the follow-up. We found no correlation between changes in CFFCP antibody titers and changes in most parameters of disease activity during follow-up. Our results are very similar to those observed with anti-CCP2 antibodies in this and other studies [42-44], but differ from those observed with anti-citrullinated vimentin antibodies, in which an association between clinical improvement and change in antibody titers was reported in early RA [40]. We do not know whether this reflects different antibody properties or differences in treatment strategies with DMARDs.

## Conclusions

In conclusion, CFFCP antibodies have a high sensitivity and specificity for RA when compared in a large series of patients with various rheumatic conditions and healthy controls, with results comparable to those obtained with the commercial CCP2 test. Anti-CFFCP1 antibodies seem to give better results than the other anti-CFFCPs and CCP2 antibodies in terms of identifying patients with poor radiographic outcome.

## Abbreviations

ACPA: anti-citrullinated protein/peptide antibodies; ACR: American College of Rheumatology; Arg: arginine; CCP1: cyclic citrullinated first-generation peptide assay; CCP2: cyclic citrullinated second-generation peptide assay; CCP3: cyclic citrullinated third-generation peptide assay; CFFCP1: chimeric fibrin/filaggrin citrullinated peptides 1; CFFCP2: synthetic chimeric fibrin/filaggrin peptides 2; CFFCP3: synthetic chimeric fibrin/filaggrin peptides 3; Cit: citrulline residue; DAS28: disease activity score in 28 joints; DMARDs: disease-modifying antirheumatic drugs; ELISAs: enzyme-linked immunosorbent assays; HCV: hepatitis C virus; mHAQ: modified Health Assessment Questionnaire; NPV: negative predictive value; PPV: positive predictive value; PsA: psoriatic arthritis; RA: rheumatoid arthritis; RF: rheumatoid factor; SLE: systemic lupus erythematosus; TNF: tumor necrosis factor.

## Competing interests

The CFFCP test is currently in process for a patent. We have not received any reimbursements, fees, funding, or salary from any organization.

## Authors' contributions

RS had full access to all the data in the study and takes responsibility for the integrity of the data and the accuracy of the data analysis. RS, JDC, and IH designed the study. RS, EG, JAG-P, MLP, and MJG acquired the data. RS, EG, IH, OV, GE, JG, ML, and AB analyzed and interpreted the data. RS, IH, ML, JAG-P, JDC, and AB prepared the manuscript. RS and JG performed statistical analyses.
